# Insights into projected changes in marine heatwaves from a high-resolution ocean circulation model

**DOI:** 10.1038/s41467-020-18241-x

**Published:** 2020-08-28

**Authors:** Hakase Hayashida, Richard J. Matear, Peter G. Strutton, Xuebin Zhang

**Affiliations:** 1 Institute for Marine and Antarctic Studies, University of Tasmania, Hobart, Tasmania Australia; 2 Australian Research Council Centre of Excellence for Climate Extremes, University of Tasmania, Hobart, Tasmania Australia; 3 CSIRO Oceans and Atmosphere, Hobart, Tasmania Australia; 4 Centre for Southern Hemisphere Oceans Research, CSIRO Oceans and Atmosphere, Hobart, Tasmania Australia

**Keywords:** Environmental sciences, Environmental impact, Ocean sciences, Physical oceanography

## Abstract

Global climate models project the intensification of marine heatwaves in coming decades due to global warming. However, the spatial resolution of these models is inadequate to resolve mesoscale processes that dominate variability in boundary current regions where societal and economic impacts of marine heatwaves are substantial. Here we compare the historical and projected changes in marine heatwaves in a 0.1° ocean model with 23 coarser-resolution climate models. Western boundary currents are the regions where the models disagree the most with observations and among themselves in simulating marine heatwaves of the past and the future. The lack of eddy-driven variability in the coarse-resolution models results in less intense marine heatwaves over the historical period and greater intensification in the coming decades. Although the projected changes agree well at the global scale, the greater spatial details around western boundary currents provided by the high-resolution model may be valuable for effective adaptation planning.

## Abstract (web summary)

Marine heatwaves are likely to intensify in a warmer world, but prediction of these events is hampered by course-scale modeling. Here the authors develop a fine scale, global model which shows that marine heatwaves will amplify with greater spatial variability, particularly at western boundary regions.

## Introduction

Satellite observations and in situ measurements of sea surface temperature (SST) indicate an increase in the frequency, duration, and intensity of marine heatwaves (MHWs) in the global ocean over the last two decades, owing largely to an increase in mean SST but also to shifts in SST variability^1^. The intensification of MHWs is a concern for marine organisms that are close to exceeding their thermal tolerance levels. MHWs can also increase the likelihood of ecosystem changes through mechanisms other than thermal stress. Reported ecological and economic impacts of recent MHW events include local extinction of mangrove and kelp forests^2^, coral bleaching^3–5^, elevated mortalities of invertebrates^6^, fishes, seabirds^7^, and marine mammals, and invasions of non-native species^8^. These changes have altered global biodiversity and caused societal impacts^9^.

The observed increasing trend in MHWs is expected to continue through this century globally, based on projections of global climate models participating in the Coupled Model Intercomparison Project 5 (CMIP5)^10,11^. Although these models are useful in assessing the impacts of ongoing climate change on MHWs at the global scale, the spatial resolution of many of these models is too coarse to resolve mesoscale processes that play a substantial role in the dynamics of the ocean^12^. In particular, western boundary currents are regions of intense eddy activity where high-resolution models simulate the historical mean state and variability better than the coarse-resolution models^13^. This includes the simulation of MHWs^14^. Therefore, the projected changes in MHWs in western boundary current regions may be better represented in higher-resolution models.

Here we investigate historical and projected changes in MHW characteristics over 1982–2050 as simulated by a dynamically downscaled near-global (75°S–75°N) ocean circulation model (OFAM3^15–17^; see “Methods”). The spatial resolution of the model (0.1°) is much finer than the typical resolution (1° for the ocean model components^18^) of global climate models participating in CMIP5^19^. At this resolution, the model resolves mesoscale eddies^12^, realistic boundary currents and fronts^15–17,20^, and is therefore superior to coarse-resolution models for studying, among other phenomena, coastal impacts where important marine resources exist^17,21^. The results of the high-resolution model simulation are compared directly with those of an observation-based daily SST analysis product of the Japan Meteorological Agency (MGD)^22^ as well as the multi-model mean product of 23 global climate models (CMIP5).

## Results

### Historical marine heatwaves over 1982–2018

We consider two metrics for MHWs: annual MHW days (the number of MHW days per year) and mean MHW intensity (the mean temperature anomaly during all MHWs in each year relative to the seasonal climatology). For globally important habitat-forming organisms, annual MHW days alone was strongly and significantly correlated with increased coral bleaching, decreased seagrass density, and decreased kelp biomass in an observational study^9^. Furthermore, the number of annual MHW days showed a more robust correlative relationship than other common measures of ocean temperature such as mean and maximum SST^9^, and is, therefore, a key metric for assessing these kinds of ecological impacts.

**Fig. 1:**
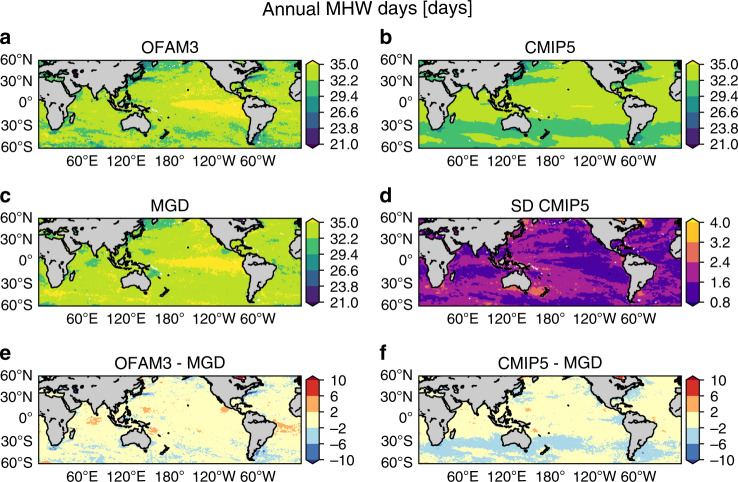
Historical annual marine heatwave days. Spatial distributions of annual marine heatwave (MHW) days averaged over 1982–2018 based on **a** a high-resolution ocean model (OFAM3), **b** the multi-model mean product of 23 global climate models (CMIP5), and **c** observations (MGD). **d** Inter-model spread of the CMIP5 models, as determined by standard deviations. Biases in **e** OFAM3 and **f** the CMIP5 multi-model mean product, as determined by their difference from MGD.

**Fig. 2:**
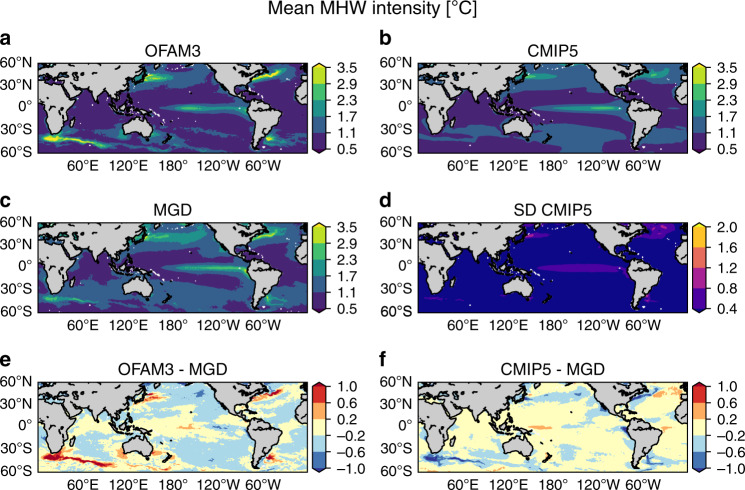
Historical mean marine heatwave intensity. Spatial distributions of mean marine heatwave (MHW) intensity averaged over 1982–2018 based on **a** a high-resolution ocean model (OFAM3), **b** the multi-model mean product of 23 global climate models (CMIP5) and **c** observations (MGD). **d** Inter-model spread of the CMIP5 models, as determined by standard deviations. Biases in **e** OFAM3 and **f** the CMIP5 multi-model mean product, as determined by their difference from MGD.

To evaluate model performance, simulated historical MHW metrics are compared with observations (MGD). We focus on the 60°S–60°N spatial comparison of the climatological state during the overlap period among OFAM3, CMIP5, and MGD (1982–2018; Figs. 1 and 2). Simulated and observed annual MHW days exhibit low spatial variability (mostly around 30 days) with relatively high values (>35 days) in the equatorial Pacific (Fig. 1a–c). CMIP5 shows much smoother spatial distribution than OFAM3 and MGD, with small inter-model spread (mostly <3 days; Fig. 1d). Both OFAM3 and CMIP5 show <10 fewer MHW days than MGD in western boundary currents (Fig. 1e, f).

The spatial patterns of simulated and observed historical mean MHW intensity are characterised by more intense MHWs in western boundary currents and lower intensity in the tropics and subtropical gyres except for the equatorial Pacific (Fig. 2a–c). Although these spatial patterns are in close agreement between the models and observations, the magnitude of mean MHW intensity differs considerably in western boundary currents (Fig. 2e, f). OFAM3 simulates >1 °C more intense MHWs in western boundary currents, whereas CMIP5 simulates <1 °C less intense MHWs.

**Fig. 3:**
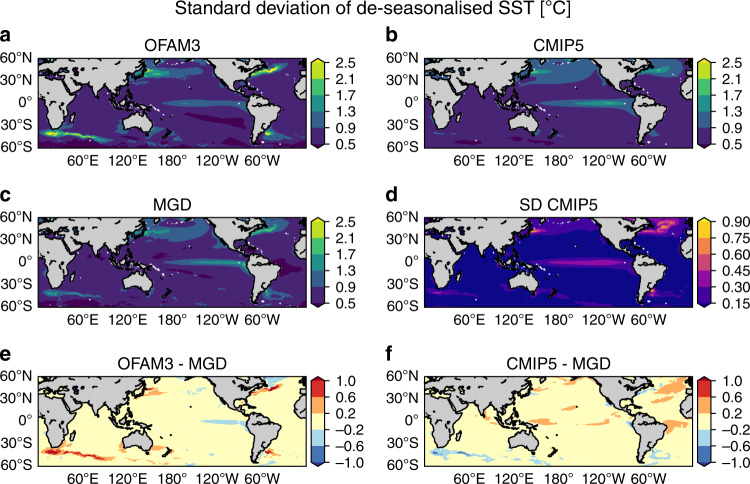
Historical standard deviation of de-seasonalised daily sea surface temperature. Spatial distributions of standard deviation of de-seasonalised daily sea surface temperature (SST) averaged over 1982–2018 based on **a** a high-resolution ocean model (OFAM3), **b** the multi-model mean product of 23 global climate models (CMIP5) and **c** observations (MGD). **d** Inter-model spread of the CMIP5 models, as determined by standard deviations. Biases in **e** OFAM3 and **f** the CMIP5 multi-model mean product, as determined by their difference from MGD.

The spatial patterns of mean MHW intensity resemble those of the standard deviation of de-seasonalised daily SST (Fig. 3), which is indicative of temporal variability at the scale relevant for mesoscale processes and MHWs. This high correlation in the spatial patterns between these two variables is consistent with the findings of previous studies using different observational and model data products^9^. Similar to the mean MHW intensity, OFAM3 exhibits higher SST variability relative to MGD primarily around western boundary currents, whereas CMIP5 shows lower variability. While the higher values in OFAM3 may suggest an overestimated variability in these eddy-rich regions, we believe that the actual biases are smaller than indicated here. Previous studies suggest systematic negative biases of SST variability represented in gridded observation-based SST analysis products^13,23,24^. Specifically, these studies report a systematic underestimate of eddy kinetic energy in the Southern Ocean by as much as 60–70% when calculated from gridded altimetry data because of interpolation, which smooths variability, compared to along-track data. Given that the SST analysis products such as MGD are also optimally interpolated, the SST variability, and hence the mean MHW intensity calculated from these gridded products may likewise be underestimated. This implies that the positive biases of OFAM3 are likely smaller and the negative biases of CMIP5 are larger than the ground truth. Another factor in these differences is the spatial resolution, which determines the variability due to fine-scale features that can be captured in each product. In this aspect, the higher variability of OFAM3 and the lower variability of CMIP5 relative to MGD are expected results.

### Projected changes over the next three decades

**Fig. 4:**
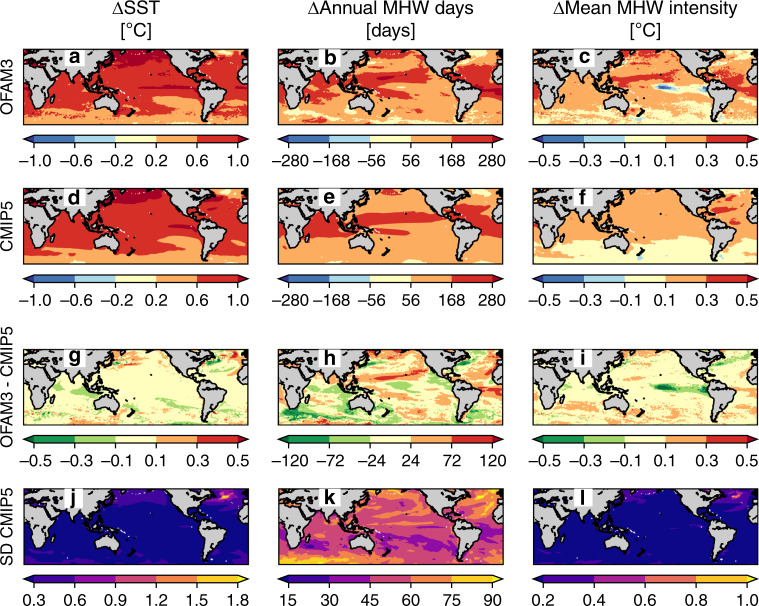
Projected changes in sea surface temperature and marine heatwaves. Spatial distributions of projected changes (2021–2050 minus 1982–2018) in mean sea surface temperature (SST), annual marine heatwave (MHW) days, and mean MHW intensity based on **a–c** a high-resolution ocean model (OFAM3) and **d–f** the multi-model mean product of 23 global climate models (CMIP5) under the Representative Concentration Pathway 8.5, **g–i** the difference between the two products (OFAM3 minus CMIP5), and **j–l** the inter-model spread of the CMIP5 models, as determined by standard deviations.

Projected changes in mean SST, annual MHW days, and mean MHW intensity under the highest-emission Representative Concentration Pathway 8.5 (RCP8.5) scenario^25^ are compared between OFAM3 and CMIP5 (Fig. 4). These changes represent the difference between the simulated climatological state over the recent decades (1982–2018) and the next 30 years (2021–2050). In terms of the 60°S–60°N global averages, the projected changes agree well between OFAM3 and CMIP5: an increase of 0.75 °C in mean SST (for both OFAM3 and CMIP5), an increase of 149 days (OFAM3) vs. 144 days (CMIP5) in annual MHW days, and an increase of 0.17 °C (OFAM3) vs. 0.16 °C (CMIP5) in mean MHW intensity (Fig. 4a-f). Therefore, OFAM3 and CMIP5 experience the same level of SST warming and nearly the same level of MHW intensification at the global scale.

The spatial patterns of projected mean SST increase are consistent between OFAM3 and CMIP5, depicted by >1 °C warming in the subpolar North Pacific, equatorial Pacific, and parts of the northern North Atlantic (Fig. 4a, d). The rate of warming is lower in the subpolar North Atlantic and to a lesser extent in the Southern Ocean. The difference in the rate of warming between OFAM3 and CMIP5 is generally larger in western boundary currents (Fig. 4g). Notably, the OFAM3-projected warming is about 0.5 °C lower in the Kuroshio Current and Gulf Stream than the CMIP5-projected warming. The inter-model spread of the CMIP5 SST projections is highest in the subpolar North Atlantic (Fig. 4j).

Both OFAM3 and CMIP5 project a greater increase in annual MHW days in many parts of the tropics and subtropical gyres (except for the equatorial Pacific) and subpolar North Pacific (Fig. 4b, e). In contrast, the rate of increase is much less in the subpolar North Atlantic and near 60 °S. In addition, OFAM3 projects similarly low rates of increase in the western boundary currents, central equatorial Pacific, Leeuwin Current, and Antarctic Circumpolar Current, but these features are absent in CMIP5. Consequently, the projected increase in annual MHW days in these regions is substantially (>120 days in some cases) lower in OFAM3 than CMIP5 (Fig. 4h). Conversely, OFAM3 projects notably higher increase in annual MHW days in the subpolar North Pacific, northeast North Atlantic, subtropical gyres, and the south of the Indian sector of the Antarctic Circumpolar Current. The inter-model spread of CMIP5 is relatively high in some parts of the Southern Ocean, subpolar North Pacific, and North Atlantic (Fig. 4k).

Projected changes in mean MHW intensity common to both OFAM3 and CMIP5 include greater intensification in the subtropical North Atlantic and lesser intensification and weakening in some cases in the subpolar North Atlantic and Southern Ocean (Fig. 4c, f).

There are several regions of large differences in the projected changes in mean MHW intensity between OFAM3 and CMIP5 (Fig. 4i). OFAM3 projects greater intensification in some parts of the North Pacific, East Australian Current (including the South of Tasmania), and Brazil Current. Furthermore, the OFAM3-projected mean MHW intensity exhibits a decrease in the central and eastern equatorial Pacific, Kuroshio Current, and Gulf Stream. The inter-model spread of CMIP5 is relatively large in the subpolar North Atlantic (Fig. 4l), consistent with the spread for SST and annual MHW days discussed above (Fig. 4j, k).

### Spatial details in western boundary currents

**Fig. 5:**
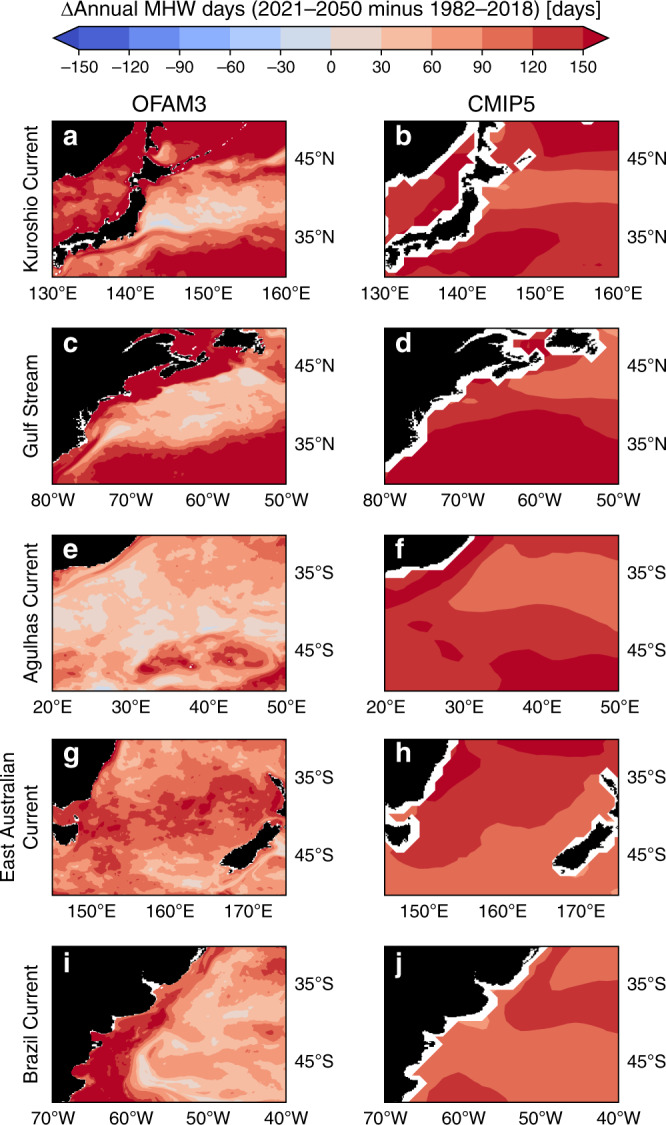
Projected marine heatwave days in western boundary current regions. Spatial comparisons of projected changes (2021–2050 minus 1982–2018) in annual marine heatwave (MHW) days in **a**, **b** Kuroshio Current, **c**, **d** Gulf Stream, **e**, **f** Agulhas Current, **g**, **h** East Australian Current and **i**, **j** Brazil Current regions between the high-resolution (0.1°) ocean model output (OFAM3; left column) and the 1° multi-model mean product of 23 global climate models (CMIP5; right column) under the Representative Concentration Pathway 8.5.

**Fig. 6:**
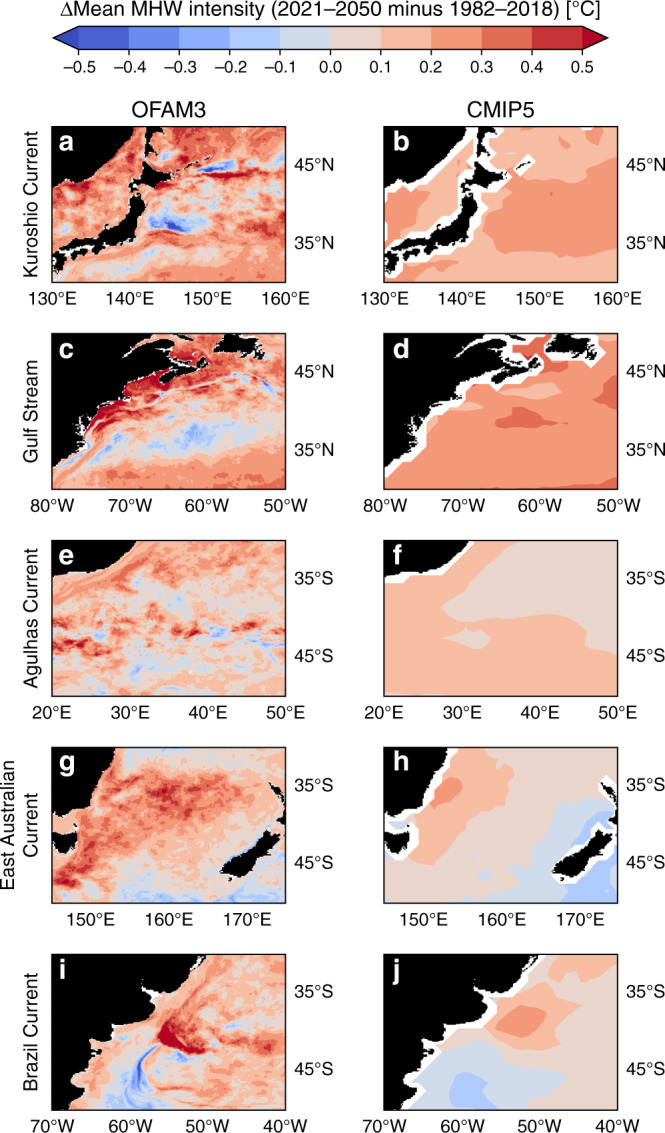
Projected marine heatwave intensity in western boundary current regions. Spatial comparisons of projected changes (2021–2050 minus 1982–2018) in mean marine heatwave (MHW) intensity in **a**, **b** Kuroshio Current, **c**, **d** Gulf Stream, **e**, **f** Agulhas Current, **g**, **h** East Australian Current, and **i**, **j** Brazil Current regions between the high-resolution (0.1°) ocean model output (OFAM3; **left column**) and the 1° multi-model mean product of 23 global climate models (CMIP5; **right column**) under the Representative Concentration Pathway 8.5.

As demonstrated by the global map comparison (Fig. 4h, i), western boundary currents stand out as the regions of disagreement between OFAM3 and CMIP5 in MHW projections. We now focus on the spatial patterns of the projected changes in each of the five western boundary currents in the 0.1° OFAM3 output, and compare them with those of the 1° CMIP5 output (Figs. 5 and 6).

Both OFAM3 and CMIP5 generally agree in the spatial patterns of projected changes in annual MHW days and mean MHW intensity, but the former provides much more spatial details, including elevated changes along the tracks of boundary currents. For example, in OFAM3, the pathway of the Kuroshio Current along the southern coast of Japan is depicted by a more pronounced increase in annual MHW days than its surroundings (Fig. 5a). Resolving such spatial variability will be essential for predicting bluefin tuna recruitment^26^. Another notable example is in the projected mean MHW intensity change along the track of the Brazil Current (Fig. 6i, j). While both OFAM3 and CMIP5 show an increase in the northern part and a decrease in the southern part of the domain, the former exhibits much finer spatial structures with higher variability.

Importantly, the 0.1° product can provide information on the projected changes along coastal areas, which is impossible with the 1° product as indicated by missing values in white. Dynamics in these narrow regions can be quite different from those of the offshore areas, and so the projected changes can likewise be different. For example, the southeast coast of Tasmania experiences a negligible increase in mean MHW intensity (<0.1 °C), whereas its offshore counterpart shows much greater increase (Fig. 6g). Hence, the higher-resolution model output such as ours can be used for risk assessment of temperature-sensitive marine aquaculture, such as Pacific oyster farming^27^.

### Relationship with sea surface temperature warming

**Fig. 7:**
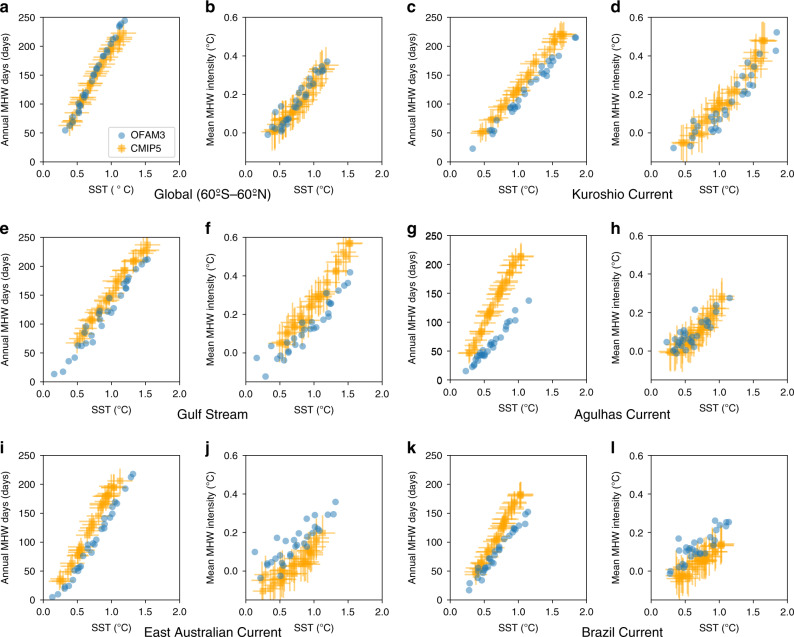
Relationship between marine heatwaves and sea surface temperature. Spatially averaged projected changes in marine heatwave (MHW) metrics vs. sea surface temperature (SST) during 2021–2050 simulated by the 0.1° ocean model (OFAM3) and the multi-model mean product of 23 global climate models (CMIP5) under the Representative Concentration Pathway 8.5. Circles and squares represent anomalies in annual mean MHW days, mean MHW intensity, and annual mean SST relative to their 1982–2018 averages over **a**, **b** the global ocean (60°S–60°N), **c**, **d** Kuroshio Current, **e**, **f** Gulf Stream, **g**, **h** Agulhas Current, **i**, **j** East Australian Current and **k**, **l** Brazil Current. The spatial domains of these western boundary current regions are defined in Fig. 5. Error bars denote the inter-model spread of the CMIP5 models, as determined by standard deviations.

The relationship between projected MHW intensification and SST warming is examined both at the global and regional scales (Fig. 7). Both OFAM3 and CMIP5 show that annual MHW days and mean MHW intensity increase linearly with mean SST warming in the next three decades globally as well as in the western boundary current regions. This finding is expected because we use a fixed baseline period (1982–2018) to define MHWs, but is useful to quantify such a relationship for assessing ecological impacts^9^.

Both the OFAM3 and CMIP5 projections indicate that the global- and regional-mean SSTs in western boundary currents will be at least 1 °C warmer in the next three decades under the RCP8.5 scenario than their averages over 1982–2018. At this level of warming, the number of simulated annual MHW days increases by ~200 days and the mean MHW intensity increases by nearly 0.3 °C on global average (Fig. 7a, b). Regionally, the same linear relationship holds, but reveals different slopes (Fig. 7c–l). Notably, the rates of increases in annual MHW days and mean MHW intensity are generally lower (roughly 150 days and 0.15 °C increases at 1 °C warming) in the western boundary current regions than those of the global averages. Among these western boundary currents, the Agulhas Current is where OFAM3 and CMIP5 differs the most in terms of the rate of increase in annual MHW days (roughly 100 vs. 200 days per 1 °C warming; Fig. 7g). The linear relationship with mean SST warming is noisier for mean MHW intensity than annual MHW days, in which OFAM3 reveals more variability than CMIP5, presumably due to the multi-model averaging of CMIP5.

## Discussion

Western boundary currents are regions of fast and variable currents, where a substantial fraction of MHWs are generated by internal variability arising from local forcing and mesoscale processes, rather than large-scale climate modes^28^. Higher spatial resolution models outperform their lower resolution counterpart in simulating MHWs of recent decades^14^ because they simulate realistic mean flow and mesoscale variability^13^. Our study demonstrates that coarse-resolution global climate models simulate less intense MHWs in western boundary current regions due to the lack of strong internal variability. Although our high-resolution ocean model considered here may have overestimated the mean intensity of MHWs by overestimating internal variability, the magnitude of this bias is smaller than it appears because of plausible negative biases of SST variability in gridded observation-based products. For a better assessment of model performance, future studies may address this issue as similarly done for sea surface height^23^.

The internal SST variability plays an important role in shaping the projected increase in annual MHW days at the regional scale. Our high-resolution ocean model results suggest that the strong internal variability of western boundary currents, the Leeuwin Current, and the equatorial Pacific, alleviates a substantial increase in annual MHW days due to the mean SST warming. As a result, the probability of MHW occurrence in these regions remains relatively unchanged compared to the rest of the global ocean. In contrast, the results based on the coarse-resolution global climate models do not show such a spatial pattern. Given that the global-scale SST warming and MHW intensification projected by the high-resolution and coarse-resolution models are nearly identical, these regional-scale differences highlight the impact of resolved ocean dynamics on the distribution of heat absorbed by the ocean under anthropogenic global warming.

By construction, our model projection assumes no change in the interannual variability in the atmospheric forcing. Projected changes in MHWs are driven primarily by global warming shifting the mean state of ocean temperature, which we believe to be robust as previously demonstrated using a statistical model for the recent past^29^ and CMIP5 model projections^10^. However, it would be worthwhile to investigate the impacts of projected changes in local air-sea coupled feedback^30,31^, climate modes^32,33^, and extremes^34^ on MHWs, which are not addressed here because of the way the future atmospheric forcing is developed. This can be achieved in a future study by: (1) prescribing the projected atmospheric conditions of a climate model projection; and (2) using a moving baseline period for the MHW definition^35^ instead of the fixed historical baseline period used here and in previous MHW projection studies^10,11,36^. Such an approach would be useful for assessing impacts on organisms that can adapt to rapidly emerging warmer mean temperatures but are vulnerable to extreme events. Another important assumption is that our results, which are based on the future projection driven by the ensemble average of CMIP5 models, depict a reasonable representation of the future ocean. Although ensemble projection at 0.1° resolution is not feasible now, such an approach would be necessary to fully assess the uncertainty in the projected MHWs.

In this study, we investigate the projected changes in MHWs using a high-resolution (0.1°) ocean circulation model. Overall, the spatial patterns of the MHW projection in the next three decades are consistent between the high-resolution ocean model and the multi-model product of the coarse-resolution global climate models at the global scale. Yet, there are substantial differences around western boundary current systems and other high-flow regions such as the Antarctic Circumpolar Current and the Leeuwin Current. High-resolution models provide more realistic representation of eddy-driven heat transport near the coast^13,15,16,20,37,38^ where the impacts of MHWs on our society and economy are greatest. Therefore, high-resolution output may provide valuable information for effective risk assessment and adaptation planning. To what extent the spatial resolution of ocean models needs to be increased to adequately assess the impacts of MHWs remains an active research question.

## Methods

### OFAM3 model description and simulation setup

We analyse the daily SST output of two simulations (historical over 1982–2005 and future projection over 2006–2050) conducted using the Ocean Forecasting Australian Model version 3 (OFAM3)^15^ developed by the CSIRO Ocean Downscaling Strategic Project. OFAM is an eddy-rich (0.1°) near-global (75°S–75°N) configuration of the Modular Ocean Model version 4.1 (MOM4p1)^39^. There are 51 non-uniform vertical layers, with the resolution of 5 m in the uppermost layer and increasing with depth. The model is initialized from rest and with the temperature and salinity climatology fields of the Commonwealth Scientific and Industrial Research (CSIRO) Atlas of Regional Seas 2009 (CARS2009)^40^, and is spun up for two decades forced with the 6-hourly 0.5625° Japanese 55-year atmospheric reanalysis (JRA-55)^41^ for 1979 repeatedly (no interannual variability)^16^. The historical simulation is initialized with the final state of the 20-year spin-up, and is conducted for 36 years (1979–2014) forced with JRA-55. The future projection is initialized with the final state of the year 2005 of the historical simulation, and is conducted for 96 years (2006–2101) forced with the daily-to-interannual component of JRA-55 over 1981–2012 repeatedly three times (2006–2037, 2038–2069, and 2070–2101) with an added long-term climate change signal from the following 17 CMIP5 models under the RCP8.5 scenario: ACCESS1-0, ACCESS1-3, BNU-ESM, CanESM2, CNRM-CM5, CSIRO-Mk3-6-0, GFDL-CM3, GFDL-ESM2G, GFDL-ESM2M, GISS-E2-H, IPSL-CM5A-MR, MIROC5, MIROC-ESM-CHEM, MIROC-ESM, MPI-CGCM3 and NorESM1-M^16,17^. Given the computational expense, it is not feasible at this time to perform multiple projections. However, the model does contain chaotic behaviour due to resolving eddies, which provides a more realistic estimate of SST variability for determining the occurrence of MHWs. The highest carbon-emission RCP8.5 scenario is chosen over other RCP scenarios as it allows us to study the oceanic response to a wider range of global warming levels^42^. As the model does not simulate sea ice (it is prescribed to be consistent with the CMIP5 multi-model mean), we focus on the analysis over the extra-polar global ocean (60°S–60°N).

### MHW definition

We define MHWs following a quantitative definition developed to facilitate comparisons among studies^43^. An anomalous warming event is defined as a MHW when daily SST exceeds the 90^th^ percentile based on a long-term smoothed historical time series for five consecutive days or more. A percentile threshold approach is better than setting an absolute value for large-scale analysis, because the latter varies considerably by region^43^. The 90^th^ percentile is determined based on the model output over 1982–2018. Using a fixed historical baseline for MHW projection analyses is consistent with the previous global MHW projection studies^10,11^, and is useful for assessing impacts on marine organisms that are both vulnerable to present-day MHWs and incapable of adapting to rapidly emerging warmer mean temperatures. We define the historical period to be 1982–2018 which is the overlap period between the simulation of OFAM3 (1979–2005 for historical; 2006–2101 for RCP8.5) and CMIP5 (1850–2005 for historical; 2006–2100 for RCP8.5) and the temporal coverage of the observations (MGD SST; 1982–2018; see below). The 90th percentile and the daily climatology (for defining MHW metrics; see below) are calculated for each calendar day by incorporating daily SSTs within an 11-day window and by applying a 31-day moving average. When MHW events are separated by one or two days, they are considered as a continuous event. While the recommended threshold is the 90^th^ percentile^43^, some studies have used other percentile ranks^10,36^ or some absolute values^44^. Consistent with the previous study on global MHWs^10^, we find that changing the percentile threshold systematically shifts the numbers, and hence does not change the conclusion about the intensification of MHWs in the future projection (not shown). For quantification of MHW characteristics, we consider the following metrics: annual MHW days and mean MHW intensity. Annual MHW days are defined as the number of days that are considered MHWs in each year. Mean intensity is defined as the average of temperature anomalies during all MHW events in each year.

### Observation-based MGD SST data

To examine model performance over the historical period, we analyse the Merged satellite and in situ data Global Daily Sea Surface Temperature (MGD SST)^22^ over 1982–2018. This 0.25° global data product is based on an algorithm incorporating infra-red and microwave sensors and calibration using in situ measurements^45^. MGD SST is representative of SST at foundation depth (roughly 1–5 m)^46^, which is more comparable to OFAM3 (at 2.5 m)^15^ than the other existing products such as the National Oceanic and Atmospheric Administration Optimum Interpolation Sea Surface Temperature version 2 (which is at ~0.5 m)^47^. In addition, MGD is superior among the foundation-depth SST products in its temporal coverage (available since 1982)^45^.

### CMIP5 data

We analyse the daily-mean SST output of the historical simulation and the RCP8.5 projection of 23 CMIP5 models: ACCESS1-0, ACCESS1-3, BCC-CSM1-1, CanESM2, CCSM4, CMCC-CESM, CMCC-CM, CMCC-CMS, CNRM-CM5, GFDL-CM3, GFDL-ESM2G, GFDL-ESM2M, INMCM4, IPSL-CM5A-LR, IPSL-CM5A-MR, IPSL-CM5B-LR, MIROC-ESM, MIROC-ESM-CHEM, MIROC5, MPI-ESM-LR, MPI-ESM-MR, MRI-CGCM3, MRI-ESM1. These models are selected based on the availability of daily-mean SST of the ensemble member *r1i1p1* for the periods of 1982–2005 (historical) and 2006–2050 (RCP8.5). We calculate MHW metrics for each model on its native grid, and then interpolate the output to a regular 1° by 1° grid for calculation of multi-model averages.

## Supplementary information

Peer Review File

## Data Availability

MGD SST is available from https://www.data.jma.go.jp/gmd/goos/data/pub/JMA-product/mgd_sst_glb_D/, and the CMIP5 data are available from https://esgf-node.llnl.gov/search/cmip5/. The OFAM3 output can be made available from the corresponding author upon request.

## Code availability

The code used to detect MHWs is archived at 10.5281/zenodo.3240201, which is a modified version of the MHW detection code written by Eric C. J. Oliver (https://github.com/ecjoliver/marineHeatWaves).
