# Supplementary material for: Insights into projected changes in marine heatwaves from a high-resolution ocean circulation model
Source: Nat Commun. 2020 Aug 28;11:4352. doi: 10.1038/s41467-020-18241-x (PMC7455734; doi:10.1038/s41467-020-18241-x)
Supplement: Supplementary file 1 — Peer Review File [file 41467_2020_18241_MOESM1_ESM.docx]

Peer Review Information

**Journal:** Nature Communications

**Manuscript title:** Insights into projected changes in marine heatwaves from a high-resolution ocean circulation model

**Corresponding author name(s):** Dr Hakase Hayashida

**Editorial notes:**

**none**

**Reviewer comments & decisions:**

**Reviewer comments, first version:**

Reviewers' comments:

Reviewer #1 (Remarks to the Author):

This manuscript reports on an analysis of output from a set of eddy-resolving global ocean model simulations forced with both historical and future atmospheric conditions. The focus in this work is marine heatwave statistics, and as the title notes one feature highlighted is the time of emergence for the future temperature range to exceed that of the historical reference period. The novelty in this work is the use of the high-resolution ocean model, which the authors argue is valuable for its ability to more faithfully simulate coastal ocean processes.

I find this manuscript to be very well written, the methods mostly easy to follow (but see my comments below about some aspects of the presentation), and the results to be interesting but not novel or surprising based on previously published work. As noted here, a 2018 study (Ref 12) serves as a model for this study questions addressed, with very similar figures, statistics, and comparisons between past and future simulated SST fields. The key differences between that study and this one is in the use of CMIP5 earth system models with coarser resolution ocean components in the former, and the prescribed atmospheric forcing derived from CMIP5 models and a historical reanalysis used as forcing for the future ocean model simulation (at higher spatial resolution) in the latter. These two studies also used different criteria for defining MHW events, but that doesn’t appear to have a major impact on the main results of either study.

While the authors note that the higher resolution here is important for resolving nearshore ocean dynamics and features, the results of this work are presented as either global maps for globally aggregated statistics. For this reason, I think the article does not make a strong case for the value of the high-resolution ocean model. Another weakness of this work, I think, is the way that ecosystem impacts are related to various global mean temperature changes and related changes in MHW statistics. As I have noted in my specific comments below, there are several passages that seem intent on making an argument for substantial marine life impacts when future SST ranges no longer overlap with those of the past. Based on observed marine life impacts of recent episodic MHW events, I think it is pretty clear that there are and will be dramatic marine life impacts well-before that level of warming is reached for many species and locations.

I think this manuscript would benefit from major revisions that provide more detailed analyses of the nearshore/coastal marine ecosystems that are better resolved by the high-resolution ocean model. A focus on coastal systems would then narrow the scope of this work to the most novel dimensions of the approach used here. I think many readers would like to know if the increased resolution provides new insights into future scenarios for ocean temperatures in these critical regions.

This manuscript also notes that the basic mechanism at work for changing MHW statistics (as defined here) in the global oceans is the underlying warming trend. The modeling approach used here could advance our understanding for whether or not, and how, ocean dynamics might modify the coastal ocean’s thermal response to anthropogenic global warming. This would be an opportunity to more carefully evaluate the extent to which high-resolution ocean model’s alter the ocean’s response to the same global-scale radiative forcing changes.

Finally, the manuscript makes use of a definition of MHWs that leads to major changes due to smooth warming trends, rather than any necessary changes to episodic events. At the least, I think this work would benefit from some discussion of Jacox’s (2019) commentary about MHWs in a changing climate where he correctly points out that for an event based analysis, one would have to update the reference period temperature to track warming trends as they evolve in time.

See Jacox, 2019: MHWs in a changing climate. Nature 571, 485-487 (2019)
doi: 10.1038/d41586-019-02196-1

Additional comments:

21: “regime shift” terminology has some baggage in fisheries oceanography, and I think you should use other terminology to signify the “new thermal range” or “exceeding the historical envelope of temperature variations” to be more specific

33-34: that marine ecosystems might have thermal tolerances is an interesting idea, but isn’t it more accurate to say that different species or species-groups have different thermal tolerances, rather than ecosystems?

45: What does it mean to “induce a regime shift … in MHWs”? Warming trends of a magnitude that move the “current” temperature range beyond the historical range? Your Figure 4 shows that changes in MHW statistics mostly follow global mean temperature change in a linear fashion, rather than some abrupt shift (outside of MHW area at the low end of warming). I think you should drop the “regime shift” terminology here and elsewhere.

81: Interesting that the OFAM has a warm bias relative to OISST but not MGD when the latter is a 5-10 m depth temperature. How does that work? I would expect that the 0.5m OISST would have a warm bias relative to MGD and OFAM (5m temperature).

102-103: Once the annual number of MHW days reaches 365, it cannot increase at all! I think you can delete this last sentence

120: I’m not sure what you mean by “choose 20 year segments to filter the inter annual variability. Your simulations involve running the same “high-frequency” JRA-55 atmospheric forcing sequences drawn from the 1981-2012 period three times, added onto the 20-year running average of the CMIP5 ensemble atmospheric forcing. Each 20 year segment selected for future periods experiences the same interannual variability by design. Notably, the historical forcing sequence contains two extreme tropical El Niño events (1982-83 and 1997-98) that I suspect play a role in the spatial patterns in your projected MHW metrics. Is that role large or small? Have you checked this?

149-155: I think this is an overly simplistic and optimistic assessment of ocean warming impacts on marine life. Ecosystem impacts are likely to be very large long-before the entire temperature distribution moves to a warmer range (for instance, the kelp forest losses around Australia or Northern California, or coral bleaching of the Great Barrier Reef, that have already been documented for episodic MHWs where “current” and historical temperature ranges are still largely overlapping).

166-168: As noted above, based on observed marine ecosystem impacts of recent warm extremes, 2.0 C global warming will likely have devastating consequences for marine life in many (or most?) places.

195-197: Ref 9 shows that persistent episodic MHW events have already caused major ecosystem impacts and shifts – “permanent MHWs” are far beyond what looks to be needed for major ecosystem impacts (you note this below in lines 201-203). It seems to me that you would be warranted stating that permanent MHWs (by your criteria) would likely be devastating for marine life as we know it in affected areas.

209-211: With regard to projecting climate modes/variations, you might add that such changes are not addressed in this work because of the way the atmospheric forcing is developed for your ocean simulation.

416-417: Why compare the OFAM projected SST warming with ensemble of 26 CMIP5 models rather than the ensemble of the 17 CMIP5 simulations from which the atmospheric forcing for the OFAM simulation/scenario comes from?

434: Which collection of CMIP5 models go into this multi-mean? Why not stick with the 17 CMIP5 models used to generate your atmospheric forcing for the OFAM simulation for an apples to apples comparison?

Figure 1: Seems to me that you should compare the OFAM scenario output with the ensemble mean of only the 17 CMIP5 simulations used for creating the atmospheric forcing used in this study. Would that reduce/eliminate the warm bias of the OFAM global mean SST simulation relative to the CMIP5 ensemble shown in Fig. 1?

512-514: SI Figure 1: Shouldn’t OFAM-NOAA (panel b) show negative values or zeros everywhere (5 meter vs. 0.5 meter temperature, with OISST biased warm for the reasons stated in the text (lines 400-414)? Instead, panel b is positive across much of the domain. Said another way, shouldn’t NOAA mean SST be biased warm relative to both MGD and OFAM? Panels b and c suggest that this is not the case. In fact, panels b and c look to be identical – are they? Sorry if I’m incorrect, but visually I cannot see any differences.

Differences between panels e and f look to be very slight.

Panel k shows large differences in the number of MHW days between OFAM and NOAA – but given that the same 90th percentile criteria is used for each field, isn’t this rather surprising? The 90th percentile OFAM MHW days are based on the OFAM historical simulation, while that for OISST is based on OISST, right? Does it mean the shape of the probability distribution for OFAM is skewed toward large positive values relative to the pdf for OISST in the tropics?

524-529: SI Figure 3: This color scheme lacks contrast for me to visually distinguish differences between, say, a probability ratio of 10 and 30. Try another color scheme that has much greater contrast than what is done here. Maybe the white-yellow-orange-red palette that is used in other figures?

Nate Mantua
NOAA/SWFSC
Santa Cruz, CA USA


Reviewer #2 (Remarks to the Author):

“Marine heatwave” is a hot topic in ocean/climate research community and it is a good idea to explore its projected changes by global-warming. The authors used a high-resolution OGCM. They showed that it would be always warmer relative to the present-day climate. The paper is written well. However, as suggested in some previous works, the air-sea coupling effect may be crucial for marine heatwave. So, in order to deeply understand the mechanism of the SST variations associated with marine heatwaves, I believe that further study using a coupled model is necessary. I hope that the results presented in this study will be revisited in near future. At least, the authors should clarify the merits and the limitation of this study based on a high-resolution ocean model. In particular, the authors should compare the presented results with coarse resolution CMIP5 models more carefully to show that the ocean mesoscale processes are critical to discuss the projected changes associated with marine heatwaves. So, my recommendation is “major revision”.

1) As suggested in some previous works (e.g. Kataoka et al. 2014; Yuan and Yamagata 2014; Doi et al. 2015; Oettli et al. 2016;), the air-sea coupling effect may be crucial for marine heatwave. The authors should clarify the merits and the limitation of this study based on more careful comparison with coarse resolution coupled models such as CMIP5 model projections. The discussion in L178-179 may be not enough. For example, why could the authors find the differences only in the subpoloar North Atlantic between the two studies? How did the ocean mesoscale eddies affect that?

Kataoka, T., Tozuka, T., Behera, S. et al. On the Ningaloo Niño/Niña. Clim Dyn 43, 1463–1482 (2014) doi:10.1007/s00382-013-1961-z

Yuan, C. & Yamagata, T. California Niño/Niña. Sci. Rep (2014). 10.1038/srep04801

Doi, T., Behera, S. K., and Yamagata, T. ( 2015), An interdecadal regime shift in rainfall predictability related to the Ningaloo Niño in the late 1990s, J. Geophys. Res. Oceans, 120, 1388– 1396, doi:10.1002/2014JC010562.

Oettli, P., Morioka, Y. & Yamagata, T. A Regional Climate Mode Discovered in the North Atlantic: Dakar Niño/Niña. Sci Rep 6, 18782 (2016) doi:10.1038/srep18782

2) The authors should discuss the results from the ocean model more carefully because the bulk formula sometime can work as indirectly restoring to the atmospheric forcing via the surface turbulent heat flux. Is the modeled surface turbulent heat flux with the bulk formula really consistent with the original atmospheric forcing flux? The long-term climate change signal from CMIP5 models under RCP8.5 scenario is warming signal. So, people can easily expect that marine heatwaves would be always warmer relative to the present-day climate via the atmospheric forcing flux. Please clarify new insights by using a high-resolution ocean model.

3) It might be better to discuss seasonality of marine heatwave occurrence and its projected change. Is it active in summer season? For example, the marine heatwave off the west coast of Australia frequently occurred in austral summer (e.g. Feng et al. 2013).

Minor:
1) L391: Those SST datasets are not “reanalysis” data, but analysis data.


Reviewer #3 (Remarks to the Author):

The manuscript focuses on future predictions of marine heatwave frequency and characteristics under a business-as-usual scenario. Using projections from a high-resolution ocean model , the authors extract the timing when the present-day MHW thresholds will be exceeded all year- round, showing important spatial variability. In contrast, the implications of a limited warming are investigated, showing a significant reduction of the MHW likelihood if the Paris Agreement targets were to be followed.
The work is original and significant, with interesting results and nice figures. Focusing on the impact of the Paris Agreement is a great idea, and most of the methods and analysis are appropriate and convincing. However, the manuscript could be better motivated with more clarity in the message. Overall, the story could be more impactful with a greater focus on coastal and western boundary current (WBC) areas, and a deeper and more quantitative description of the results. I recommend publication after major revisions.

GENERAL:
• Both the abstract and the discussion are vague, with more highlight on the model resolution (which is under-exploited) and biological impacts than on the actual results. The message of the manuscript is somehow lost. The introduction does not motivate well the analysis and the partitioning between the text, caption and methods is sometimes confusing. An overall review of the manuscript to improve the flow of the story and transitions would greatly improve the article.
• As stated many times by the authors, the strength of the model they use is the high spatial resolution (eddy resolving, 0.1o). This should be more exploited, with an enhanced focus on coastal and WBC areas.
• In particular, the last paragraph of the result section “comparison with coarse resolution models” is far from being conclusive despite the expectations. The authors miss an opportunity to validate their results and explain the importance of the high-resolution.
• It is unclear why the NOAA OISST product was chosen. It seems that there is more text explaining why the differences are expected (difference in depth) than anything else. Other products might be more adequate anyway (see Fiedler et al, 2019).

Fiedler E.K., McLaren, A., Banzon, V., Brasmett, B., Ishizaki, S., Kennedy, J., Rayner, N., Roberts-Jones, J., Corlett, G., Merchant, C.J., Donlon, C. (2019) Intercomparison of long-term sea surface temperature analyses using the GHRSST Multi-Product Ensemble (GMPE) system, Rem Sens Env, 222, 18-33. https://doi.org/10.1016/j.rse.2018.12.015


DETAILED COMMENTS:
Introduction:
• L. 32: The increase in MHW frequency and duration is not only due to an increase in mean SST. Please re-read the citation.
• L. 43: The transition between thermal limits and MHWs occurring throughout the year is missing.


Results:
• L. 87-88: Then why did you choose SST products which are not comparable to the model?
• L. 93-96: Specify that you are now referring to OFAM results.
• L. 106: Reword “combat climate change”.
• L. 111: the method does actually not give much more information.
• L. 116: Can you quantify?
• L. 126: Typo with “Fig. 21”.
• L. 131: How can the correlation be so strong considering the mesoscale and inter-annual variability?
• L. 148-154: Nice result, which should be highlighted in the abstract and conclusion.
• L. 164-167: Make sure it is clear when temperature is SST or atmospheric warming.
• L. 169-183: Is the paragraph supposed to be a validation or an added-value of your model? In both cases it is not really conclusive.

Discussion:
• The first paragraph is interesting but very general.

Methods:
• L. 351: What is the spatial resolution of JRA-55?
• L. 381: Figure4, not 3.
• L. 433: This is a major assumption which deserves more details than just a reference.

Figures:
• Fig. 1: specify that the year 2034 is for OFAM only (L. 475). Lines 470-472 should be in the text rather than in the caption.
• Fig. 2: Since the advantage of the model is the resolution, you could add the results averaged in coastal areas and in WBC regions in panels a-c.
• Fig 4: Do the relationships hold everywhere or only for the global averages? Again, the strength of the model is the high resolution. You could add shading with standard deviations or averages over smaller relevant areas.


Supplementary Material:
• Fig. S3: I don’t understand how this figure helps comparing to CMIP5 model projections. Since you have the CMIP5 data, you could present a proper comparison.

**Author rebuttal, first version:**

Reviewers' comments:

Reviewer #1 (Remarks to the Author):

This manuscript reports on an analysis of output from a set of eddy-resolving global ocean model simulations forced with both historical and future atmospheric conditions. The focus in this work is marine heatwave statistics, and as the title notes one feature highlighted is the time of emergence for the future temperature range to exceed that of the historical reference period. The novelty in this work is the use of the high-resolution ocean model, which the authors argue is valuable for its ability to more faithfully simulate coastal ocean processes. 

I find this manuscript to be very well written, the methods mostly easy to follow (but see my comments below about some aspects of the presentation), and the results to be interesting but not novel or surprising based on previously published work. As noted here, a 2018 study (Ref 12) serves as a model for this study questions addressed, with very similar figures, statistics, and comparisons between past and future simulated SST fields. The key differences between that study and this one is in the use of CMIP5 earth system models with coarser resolution ocean components in the former, and the prescribed atmospheric forcing derived from CMIP5 models and a historical reanalysis used as forcing for the future ocean model simulation (at higher spatial resolution) in the latter. These two studies also used different criteria for defining MHW events, but that doesn’t appear to have a major impact on the main results of either study.  

While the authors note that the higher resolution here is important for resolving nearshore ocean dynamics and features, the results of this work are presented as either global maps for globally aggregated statistics. For this reason, I think the article does not make a strong case for the value of the high-resolution ocean model. Another weakness of this work, I think, is the way that ecosystem impacts are related to various global mean temperature changes and related changes in MHW statistics. As I have noted in my specific comments below, there are several passages that seem intent on making an argument for substantial marine life impacts when future SST ranges no longer overlap with those of the past. Based on observed marine life impacts of recent episodic MHW events, I think it is pretty clear that there are and will be dramatic marine life impacts well-before that level of warming is reached for many species and locations.

I think this manuscript would benefit from major revisions that provide more detailed analyses of the nearshore/coastal marine ecosystems that are better resolved by the high-resolution ocean model. A focus on coastal systems would then narrow the scope of this work to the most novel dimensions of the approach used here. I think many readers would like to know if the increased resolution provides new insights into future scenarios for ocean temperatures in these critical regions.

**Thanks for providing thorough and constructive review comments. We agree that narrowing the scope of this work to coastal regions would maximise the advantage of high-resolution modelling. We changed the focus of this study to western boundary currents where the largest differences exist between our model output and the CMIP5 multi-model mean.**

This manuscript also notes that the basic mechanism at work for changing MHW statistics (as defined here) in the global oceans is the underlying warming trend. The modeling approach used here could advance our understanding for whether or not, and how, ocean dynamics might modify the coastal ocean’s thermal response to anthropogenic global warming. This would be an opportunity to more carefully evaluate the extent to which high-resolution ocean model’s alter the ocean’s response to the same global-scale radiative forcing changes. 

**We appreciate this suggestion. The comparison between our model and CMIP5 reveals the regional difference in the projected warming and MHW metrics while they agree in the global averages of these quantities. This is now shown visually in Figure 4, described in detail in Section “Projected changes over the next three decades (1982-2018 vs. 2021-2050)”, and discussed in the second paragraph of the Discussion section:**

**L231: “The internal SST variability plays an important role in shaping the projected increase in annual MHW days at the regional scale. Our high-resolution ocean model results suggest that the strong internal variability of western boundary currents, the Leeuwin Current, and the equatorial Pacific, alleviates a substantial increase in annual MHW days due to the mean SST warming. As a result, the probability of MHW occurrence in these regions remains relatively unchanged compared to the rest of the global ocean. In contrast, the results based on the coarse-resolution global climate models do not show such a spatial pattern. Given that the global-scale SST warming and MHW intensification projected by the high-resolution and coarse-resolution models are nearly identical, these regional-scale differences highlight the impact of resolved ocean dynamics on the distribution of heat absorbed by the ocean under the anthropogenic global warming.”**

Finally, the manuscript makes use of a definition of MHWs that leads to major changes due to smooth warming trends, rather than any necessary changes to episodic events. At the least, I think this work would benefit from some discussion of Jacox’s (2019) commentary about MHWs in a changing climate where he correctly points out that for an event based analysis, one would have to update the reference period temperature to track warming trends as they evolve in time. 

See Jacox, 2019: MHWs in a changing climate. Nature 571, 485-487 (2019)
doi: 10.1038/d41586-019-02196-1

**As discussed in detail by Oliver et al. (2019), the definition should depend on the specific goal of the study. In our study, we use a fixed historical baseline for MHW projection for assessing impacts on marine organisms that are both vulnerable to present-day MHWs and incapable of adapting to rapidly-emerging warmer mean temperatures, as stated in the revised manuscript:**

**L67: “Using a fixed historical baseline for MHW projection analyses is consistent with the previous global MHW projection studies^10,11^, and is useful for assessing impacts on marine organisms that are both vulnerable to present-day MHWs and incapable of adapting to rapidly-emerging warmer mean temperatures.”**

**We agree that moving the baseline should be considered for projection studies, which is especially important for organisms that can adapt to rapidly-emerging warmer mean temperatures. Unfortunately, we cannot investigate this aspect using the OFAM3 model output due to the design of the future atmospheric forcing (repeating the interannual variability of JRA-55, so presumably no changes in climate modes). We incorporated this discussion into the revised manuscript as follows:**

**L222: “However, it would be worthwhile to investigate the impacts of projected changes in local air-sea coupled feedback^31,32^, climate modes^33,34^, and extremes^35^ on MHWs, which are not addressed here because of the way the future atmospheric forcing is developed. This can be achieved in a future study by: (1) prescribing the projected atmospheric conditions of a climate model projection; and (2) using a moving baseline period for the MHW definition^36^ instead of the fixed historical baseline period used here and in previous MHW projection studies^10,11,37^. Such an approach would be useful for assessing impacts on organisms that can adapt to rapidly-emerging warmer mean temperatures but are vulnerable to extreme events.**

**Oliver et al. Projected Marine Heatwaves in the 21st Century and the Potential for Ecological Impact. *Front. Mar. Sci.* 6, (2019).**

Additional comments:

21: “regime shift” terminology has some baggage in fisheries oceanography, and I think you should use other terminology to signify the “new thermal range” or “exceeding the historical envelope of temperature variations” to be more specific 

**Thanks for the explanation for the terminology “regime shift” in fisheries oceanography. Because of the substantial revisions, this term is no longer used in the revised manuscript.**

33-34: that marine ecosystems might have thermal tolerances is an interesting idea, but isn’t it more accurate to say that different species or species-groups have different thermal tolerances, rather than ecosystems? 

**The term “ecosystems” is changed to “organisms” in the revised manuscript (L33).**

45: What does it mean to “induce a regime shift … in MHWs”? Warming trends of a magnitude that move the “current” temperature range beyond the historical range? Your Figure 4 shows that changes in MHW statistics mostly follow global mean temperature change in a linear fashion, rather than some abrupt shift (outside of MHW area at the low end of warming). I think you should drop the “regime shift” terminology here and elsewhere. 

**As noted above, the term “regime shift” is no longer used in the revised manuscript.**

81: Interesting that the OFAM has a warm bias relative to OISST but not MGD when the latter is a 5-10 m depth temperature. How does that work? I would expect that the 0.5m OISST would have a warm bias relative to MGD and OFAM (5m temperature).

**We agree, but we did not further address this issue as we excluded NOAA OISST from the revised manuscript. We chose MGD SST as it is representative of foundation depth, which is more consistent with the model’s SST (at 2.5 m) than NOAA OISST (0.5 m).**

102-103: Once the annual number of MHW days reaches 365, it cannot increase at all! I think you can delete this last sentence

**The last sentence is deleted in the revised manuscript.**

120: I’m not sure what you mean by “choose 20 year segments to filter the inter annual variability. Your simulations involve running the same “high-frequency” JRA-55 atmospheric forcing sequences drawn from the 1981-2012 period three times, added onto the 20-year running average of the CMIP5 ensemble atmospheric forcing. Each 20 year segment selected for future periods experiences the same interannual variability by design. Notably, the historical forcing sequence contains two extreme tropical El Niño events (1982-83 and 1997-98) that I suspect play a role in the spatial patterns in your projected MHW metrics. Is that role large or small? Have you checked this? 

**We did not check this initially. In the revised manuscript, we changed the projected period to the next three decades (2021-2050), which corresponds to 1996-2012 and 1981-1993 of the JRA55 years. We compare the spatial patterns based on four different periods (2021-2050, 2022-2050, 2023-2050, and 2024-2050; Figure R1 below). The period 2024-2050 corresponds to 1999-2012 and 1981-1993 of the JRA55 years, therefore excludes one of the extreme El Nino events (1997-1998). As seen in Figure R1 (below), the spatial patterns of projected SST and MHW metrics appear to be insensitive to the choice of period selected here.**

**Figure R1: Comparison of projected changes in SST (left), annual MHW days (middle), and mean MHW intensity (right) among different time periods (2021-2050, 2022-2050, 2023-2050, and 2024-2050) relative to 1982-2018.**

149-155: I think this is an overly simplistic and optimistic assessment of ocean warming impacts on marine life. Ecosystem impacts are likely to be very large long-before the entire temperature distribution moves to a warmer range (for instance, the kelp forest losses around Australia or Northern California, or coral bleaching of the Great Barrier Reef, that have already been documented for episodic MHWs where “current” and historical temperature ranges are still largely overlapping).

**This section has been removed as a result of substantial revisions.**

166-168: As noted above, based on observed marine ecosystem impacts of recent warm extremes, 2.0 C global warming will likely have devastating consequences for marine life in many (or most?) places. 

**This section has been removed as a result of substantial revisions.**

195-197: Ref 9 shows that persistent episodic MHW events have already caused major ecosystem impacts and shifts – “permanent MHWs” are far beyond what looks to be needed for major ecosystem impacts (you note this below in lines 201-203). It seems to me that you would be warranted stating that permanent MHWs (by your criteria) would likely be devastating for marine life as we know it in affected areas. 

**This sentence has been removed as a result of substantial revisions.**

209-211: With regard to projecting climate modes/variations, you might add that such changes are not addressed in this work because of the way the atmospheric forcing is developed for your ocean simulation.

**As suggested, we revised the text as follows:**

**L248: “… which are not addressed here because of the way the future atmospheric forcing is developed.”**

416-417: Why compare the OFAM projected SST warming with ensemble of 26 CMIP5 models rather than the ensemble of the 17 CMIP5 simulations from which the atmospheric forcing for the OFAM simulation/scenario comes from?

**This figure is excluded from the revised manuscript. We agree that comparing with the same 17 models used for the future atmospheric forcing is ideal (allows apple to apple comparison). However, some of these model outputs were not available for daily-mean SST, which is what we used for the revised manuscript (e.g. CSIRO-Mk-3-6-0 has missing values in ice-covered regions, which includes the upper part of our 60S-60N domain; BNU-EMS and NorESM1-M are not available for the full 20060101-20501231 coverage). In the end, we compared OFAM3 with the multi-model mean of 23 CMIP5 models based on availability. Fortunately, the projected SST warming is identical (0.75 °C) between OFAM3 and the CMIP5 multi-model mean in terms of the 60°S-60°N global averages, which we describe in the revised manuscript:**

**L127: “In terms of the 60°S-60°N global averages, the projected changes agree well between OFAM3 and CMIP5: an increase of 0.75 °C in mean SST (for both OFAM3 and CMIP5), …”**

434: Which collection of CMIP5 models go into this multi-mean? Why not stick with the 17 CMIP5 models used to generate your atmospheric forcing for the OFAM simulation for an apples to apples comparison?

**The 17 models used for the future atmospheric forcing development are listed as follows:**

**L389: “… with an added long-term climate change signal from the following 17 CMIP5 models under the RCP8.5 scenario: ACCESS1-0, ACCESS1-3, BNU-ESM, CanESM2, CNRM-CM5, CSIRO-Mk3-6-0, GFDL-CM3, GFDL-ESM2G, GFDL-ESM2M, GISS-E2-H, IPSL-CM5A-MR, MIROC5, MIROC-ESM-CHEM, MIROC-ESM, MPI-CGCM3, and NorESM1-M^16,17^.”**

**And the 23 models used for the comparison of SST and MHW metrics are listed as follows:**

**L432: “CMIP5 data. We analyse the daily-mean SST output of the historical simulation and the RCP8.5 projection of 23 CMIP5 models: ACCESS1-0, ACCESS1-3, BCC-CSM1-1, CanESM2, CCSM4, CMCC-CESM, CMCC-CM, CMCC-CMS, CNRM-CM5, GFDL-CM3, GFDL-ESM2G, GFDL-ESM2M, INMCM4, IPSL-CM5A-LR, IPSL-CM5A-MR, IPSL-CM5B-LR, MIROC-ESM, MIROC-ESM-CHEM, MIROC5, MPI-ESM-LR, MPI-ESM-MR, MRI-CGCM3, MRI-ESM1. These models are selected based on the availability of daily-mean SST of the ensemble member *r1i1p1* for the periods of 1982-2005 (historical) and 2006-2050 (RCP8.5).”**

Figure 1: Seems to me that you should compare the OFAM scenario output with the ensemble mean of only the 17 CMIP5 simulations used for creating the atmospheric forcing used in this study. Would that reduce/eliminate the warm bias of the OFAM global mean SST simulation relative to the CMIP5 ensemble shown in Fig. 1? 

**This figure is excluded from the revised manuscript. As noted above, the projected SST warming in terms of global averages is identical (0.75 °C) between OFAM3 and CMIP5.**

512-514: SI Figure 1: Shouldn’t OFAM-NOAA (panel b) show negative values or zeros everywhere (5 meter vs. 0.5 meter temperature, with OISST biased warm for the reasons stated in the text (lines 400-414)? Instead, panel b is positive across much of the domain. Said another way, shouldn’t NOAA mean SST be biased warm relative to both MGD and OFAM? Panels b and c suggest that this is not the case. In fact, panels b and c look to be identical – are they? Sorry if I’m incorrect, but visually I cannot see any differences.

**We agree that NOAA should be warmer than MGD or OFAM considering the SST depth definition, but we checked and confirm that it is not the case here (and we do not know why). The difference between NOAA and MGD is very slight for all except for annual MHW days. Nevertheless, as noted above, we excluded NOAA from the revised manuscript.**

Differences between panels e and f look to be very slight.

**Again yes, the difference is very slight between NOAA and MGD here.**

Panel k shows large differences in the number of MHW days between OFAM and NOAA – but given that the same 90th percentile criteria is used for each field, isn’t this rather surprising? The 90th percentile OFAM MHW days are based on the OFAM historical simulation, while that for OISST is based on OISST, right? Does it mean the shape of the probability distribution for OFAM is skewed toward large positive values relative to the pdf for OISST in the tropics? 

**We compared skewness (Figure R2 below) but the difference is small so we do not think it is the factor here. We further investigated the difference between NOAA and MGD, and conclude that the reason for NOAA to have fewer annual MHW days is because of high frequency SST variability (Figure R3). In addition to the 90th percentile threshold, SST must be above the threshold for at least 5 days to be considered a MHW (following the standard definition of Hobday et al. 2016). As seen in Figure R3c&d, NOAA SST time series exhibit much higher variability, resulting in fewer annual MHW days. We tested the sensitivity of annual MHW days to this minimum duration definition (varying from 1 to 20 days) and find that it does have a greater impact on NOAA (Figure R3b). NOAA and MGD would have the same annual MHW days if the minimum duration was set to 2 days.**

**Nevertheless, we excluded NOAA OISST from the revised manuscript, so did not include these results in the revised manuscript. Our colleagues (Maxime Marin and others) are working on a study that compares different SST products for MHW metrics, which should address this issue in the near future.**

**Figure R2: Comparison of de-seasonalised SST skewness among MGD, NOAA, OFAM, and CMIP5.**

**Figure R3: (a) the difference in annual MHW days between NOAA and MGD; (b) the sensitivity of annual MHW days to the minimum duration defined for MHWs at the location 5 N and 155 E (indicated by x in panel A); (c) SST time series of MGD at the location; and (d) SST time series of NOAA at the location. In panels C and D, the cyan stripes indicate MHWs based on the minimum duration of 5 days (the standard definition used in this study).**

524-529: SI Figure 3: This color scheme lacks contrast for me to visually distinguish differences between, say, a probability ratio of 10 and 30. Try another color scheme that has much greater contrast than what is done here. Maybe the white-yellow-orange-red palette that is used in other figures?

**This figure is excluded from the revised manuscript. However, we appreciate your concern about colour scheme. In the revised manuscript, we used “contourf” of Python to improve the readability of numbers in the figures (as opposed to “pcolor” used in the submitted manuscript).**

Nate Mantua
NOAA/SWFSC
Santa Cruz, CA USA

**--------------------------------------------------------------------------------**

Reviewer #2 (Remarks to the Author):

“Marine heatwave” is a hot topic in ocean/climate research community and it is a good idea to explore its projected changes by global-warming. The authors used a high-resolution OGCM. They showed that it would be always warmer relative to the present-day climate. The paper is written well. However, as suggested in some previous works, the air-sea coupling effect may be crucial for marine heatwave. So, in order to deeply understand the mechanism of the SST variations associated with marine heatwaves, I believe that further study using a coupled model is necessary. I hope that the results presented in this study will be revisited in near future. At least, the authors should clarify the merits and the limitation of this study based on a high-resolution ocean model. In particular, the authors should compare the presented results with coarse resolution CMIP5 models more carefully to show that the ocean mesoscale processes are critical to discuss the projected changes associated
with marine heatwaves. So, my recommendation is “major revision”.

**Thanks for the constructive feedback. We agree to clarify the merits and limitations of the use of a high-resolution ocean model by directly comparing with CMIP5 models, and we revised the manuscript accordingly.**

1) As suggested in some previous works (e.g. Kataoka et al. 2014; Yuan and Yamagata 2014; Doi et al. 2015; Oettli et al. 2016;), the air-sea coupling effect may be crucial for marine heatwave. The authors should clarify the merits and the limitation of this study based on more careful comparison with coarse resolution coupled models such as CMIP5 model projections. The discussion in L178-179 may be not enough. For example, why could the authors find the differences only in the subpoloar North Atlantic between the two studies? How did the ocean mesoscale eddies affect that?

Kataoka, T., Tozuka, T., Behera, S. et al. On the Ningaloo Niño/Niña. Clim Dyn 43, 1463–1482 (2014) doi:10.1007/s00382-013-1961-z

Yuan, C. & Yamagata, T. California Niño/Niña. Sci. Rep (2014). 10.1038/srep04801

Doi, T., Behera, S. K., and Yamagata, T. ( 2015), An interdecadal regime shift in rainfall predictability related to the Ningaloo Niño in the late 1990s, J. Geophys. Res. Oceans, 120, 1388– 1396, doi:10.1002/2014JC010562.

Oettli, P., Morioka, Y. & Yamagata, T. A Regional Climate Mode Discovered in the North Atlantic: Dakar Niño/Niña. Sci Rep 6, 18782 (2016) doi:10.1038/srep18782

**We agree that some of the discrepancies between our forced-ocean model (OFAM3) and the CMIP5 models can be due to the lack of air-sea coupling in the former model. Obviously, we are unable to investigate the role of local air-sea coupled feedback in projected MHWs with OFAM3, but we added this important point to the revised manuscript:**

**L247: “However, it would be worthwhile to investigate the impacts of projected changes in local air-sea coupled feedback^31,32^, climate modes^33,34^, and extremes^35^ on MHWs, which are not addressed here because of the way the future atmospheric forcing is developed.”**

2) The authors should discuss the results from the ocean model more carefully because the bulk formula sometime can work as indirectly restoring to the atmospheric forcing via the surface turbulent heat flux. Is the modeled surface turbulent heat flux with the bulk formula really consistent with the original atmospheric forcing flux? The long-term climate change signal from CMIP5 models under RCP8.5 scenario is warming signal. So, people can easily expect that marine heatwaves would be always warmer relative to the present-day climate via the atmospheric forcing flux. Please clarify new insights by using a high-resolution ocean model. 

**Because we use bulk formulas, there is a heat flux related to the air temperature that provides a way to “restore” the OFAM3 ocean to the CMIP5 surface temperature. As a result, the projected SST warming follows very closely with the CMIP5 projection at the global scale (as discussed in L126 of the revised manuscript). However, at the regional scale, they differ, and we compared the spatial patterns in projected MHW changes between OFAM3 and CMIP5 (Fig. 4), and provided insights into projected changes in MHWs as follows:**

**L237: “Given that the global-scale SST warming and MHW intensification projected by the high-resolution and coarse-resolution models are nearly identical, these regional-scale differences highlight the impact of resolved ocean dynamics on the distribution of heat absorbed by the ocean under the anthropogenic global warming.”**

3) It might be better to discuss seasonality of marine heatwave occurrence and its projected change. Is it active in summer season? For example, the marine heatwave off the west coast of Australia frequently occurred in austral summer (e.g. Feng et al. 2013). 

**We show the seasonality of MHW occurrence over the historical period simulated by OFAM3 in another manuscript (Hayashida et al., accepted). Projected changes in MHW seasonality would be an interesting topic, but it would be more appropriate to use a moving baseline for the MHW definition. Using a fixed baseline (our study) will likely increase the MHW occurrence in all seasons simply due to a shift in the mean state of SST.**

**Hayashida et al. (accepted): Background nutrient concentration determines phytoplankton bloom response to marine heatwaves. Global Change Biology.**

Minor:
1) L391: Those SST datasets are not “reanalysis” data, but analysis data.

**Thanks for this correction. We refer to these datasets as “analysis” throughout the revised manuscript.**

**--------------------------------------------------------------------------------**

Reviewer #3 (Remarks to the Author):

The manuscript focuses on future predictions of marine heatwave frequency and characteristics under a business-as-usual scenario. Using projections from a high-resolution ocean model , the authors extract the timing when the present-day MHW thresholds will be exceeded all year- round, showing important spatial variability. In contrast, the implications of a limited warming are investigated, showing a significant reduction of the MHW likelihood if the Paris Agreement targets were to be followed.
The work is original and significant, with interesting results and nice figures. Focusing on the impact of the Paris Agreement is a great idea, and most of the methods and analysis are appropriate and convincing. However, the manuscript could be better motivated with more clarity in the message. Overall, the story could be more impactful with a greater focus on coastal and western boundary current (WBC) areas, and a deeper and more quantitative description of the results. I recommend publication after major revisions.

**Thanks for the positive comments on our submitted manuscript. As suggested, we have revised the manuscript substantially by comparing our high-resolution ocean model results with CMIP5 models, and changed the focus to WBC areas where the models disagree the most in projected changes in MHW metrics.**

GENERAL:
• Both the abstract and the discussion are vague, with more highlight on the model resolution (which is under-exploited) and biological impacts than on the actual results. The message of the manuscript is somehow lost. The introduction does not motivate well the analysis and the partitioning between the text, caption and methods is sometimes confusing. An overall review of the manuscript to improve the flow of the story and transitions would greatly improve the article.

**We revised the manuscript substantially, and provided more information on our actual results in the abstract. The introduction has been revised to motivate the opportunity of high-resolution modelling for MHW projection:**

**L41: “The observed increasing trend in MHWs is expected to continue through this century globally, based on projections of global climate models participating in the Coupled Model Intercomparison Project 5 (CMIP5)^10,11^. Although these models are useful in assessing the impacts of ongoing climate change on MHWs at the global scale, the spatial resolution of many of these models is too coarse to resolve mesoscale processes that play a substantial role in the dynamics of the ocean^12^. In particular, western boundary currents are regions of intense eddy activity where high-resolution models simulate the historical mean state and variability better than the coarse-resolution models^13^. This includes the simulation of MHWs^14^. Therefore, the projected changes in marine heatwaves may be better represented in higher resolution models.”**

• As stated many times by the authors, the strength of the model they use is the high spatial resolution (eddy resolving, 0.1o). This should be more exploited, with an enhanced focus on coastal and WBC areas.

**As suggested, we changed the focus of the paper to western boundary currents where we find the greatest difference between our high-resolution ocean model and CMIP5.**

• In particular, the last paragraph of the result section “comparison with coarse resolution models” is far from being conclusive despite the expectations. The authors miss an opportunity to validate their results and explain the importance of the high-resolution.

**We agree, and so did a direct comparison with the CMIP5 models in the revised manuscript.**

• It is unclear why the NOAA OISST product was chosen. It seems that there is more text explaining why the differences are expected (difference in depth) than anything else. Other products might be more adequate anyway (see Fiedler et al, 2019).

Fiedler E.K., McLaren, A., Banzon, V., Brasmett, B., Ishizaki, S., Kennedy, J., Rayner, N., Roberts-Jones, J., Corlett, G., Merchant, C.J., Donlon, C. (2019) Intercomparison of long-term sea surface temperature analyses using the GHRSST Multi-Product Ensemble (GMPE) system, Rem Sens Env, 222, 18-33. [https://doi.org/10.1016/j.rse.2018.12.015](https://protect-au.mimecast.com/s/BgLFCyoNROTP4qMNizjM4F?domain=doi.org)

**We agree. Originally, we wanted to demonstrate the difference between NOAA OISST and MGD, because the former is the most commonly used product for MHW research although the depth representation is inconsistent with models. However, we decided to exclude NOAA OISST from the revised manuscript to focus on the discussion of model comparison. A comparison study of different SST products focusing on MHW metrics is underway by our colleagues (Maxime Marin and others), so we do not need to do this anymore.**

DETAILED COMMENTS:
Introduction:
• L. 32: The increase in MHW frequency and duration is not only due to an increase in mean SST. Please re-read the citation.

**We revised this as follows:**

**L32: “owing largely to an increase in mean SST but also to shifts in SST variability^1^.”**

• L. 43: The transition between thermal limits and MHWs occurring throughout the year is missing.

**This paragraph is excluded from the revised manuscript, as we changed the focus to high- vs. coarse-resolution models. Oliver et al. (2019) investigated projected changes in MHWs throughout the year (and not just summer), so this paragraph is no longer valid.**

**Oliver et al. Projected Marine Heatwaves in the 21st Century and the Potential for Ecological Impact. *Front. Mar. Sci.* 6, (2019).**

Results:
• L. 87-88: Then why did you choose SST products which are not comparable to the model?

**We agree, and excluded NOAA OISST from the revised manuscript.**

• L. 93-96: Specify that you are now referring to OFAM results.

**This sentence is deleted as a result of substantial revisions.**

• L. 106: Reword “combat climate change”.

**This section is deleted as a result of substantial revisions.**

• L. 111: the method does actually not give much more information.

**This section is deleted as a result of substantial revisions.**

• L. 116: Can you quantify?

**This section is deleted as a result of substantial revisions.**

• L. 126: Typo with “Fig. 21”.

**This section is deleted as a result of substantial revisions.**

• L. 131: How can the correlation be so strong considering the mesoscale and inter-annual variability?

**This section is deleted as a result of substantial revisions.**

• L. 148-154: Nice result, which should be highlighted in the abstract and conclusion.

**Thanks, but this section is deleted as a result of substantial revisions.**

• L. 164-167: Make sure it is clear when temperature is SST or atmospheric warming.

**This section is deleted as a result of substantial revisions.**

• L. 169-183: Is the paragraph supposed to be a validation or an added-value of your model? In both cases it is not really conclusive.

**This section is deleted as a result of substantial revisions. As noted above, we directly compare with CMIP5 models, providing more conclusive findings in the revised manuscript.**

Discussion:
• The first paragraph is interesting but very general.

**We deleted this paragraph, as we changed the focus of our paper from permanent exceedance to western boundary currents.**

Methods:
• L. 351: What is the spatial resolution of JRA-55?

**0.5625° (Kobayashi et al. 2015). We added this info to this sentence: L338: “… the 6-hourly 0.5625° Japanese 55-year atmospheric reanalysis (JRA-55) …”.**

• L. 381: Figure4, not 3.

**We deleted this figure in the revised manuscript.**

• L. 433: This is a major assumption which deserves more details than just a reference.

**This section is deleted as a result of substantial revisions.**

Figures:
• Fig. 1: specify that the year 2034 is for OFAM only (L. 475). Lines 470-472 should be in the text rather than in the caption.

**We deleted this figure in the revised manuscript.**

• Fig. 2: Since the advantage of the model is the resolution, you could add the results averaged in coastal areas and in WBC regions in panels a-c.

**We deleted this figure. In the revised manuscript, we added figures showing the spatial patterns in western boundary currents between our high-resolution ocean model and CMIP5 models (Figs. 5 and 6).**

• Fig 4: Do the relationships hold everywhere or only for the global averages? Again, the strength of the model is the high resolution. You could add shading with standard deviations or averages over smaller relevant areas.

**We examined the relationships for the regional averages of the western boundary current regions (Fig. 7) and added a section “Relationship with sea surface temperature warming”.**

Supplementary Material:
• Fig. S3: I don’t understand how this figure helps comparing to CMIP5 model projections. Since you have the CMIP5 data, you could present a proper comparison.

**We deleted this figure in the revised manuscript. As suggested, the revised manuscript presents direct comparison with CMIP5 models.**

**Reviewer comments, second version:**

Reviewer #1 (Remarks to the Author):

The revised manuscript is much improved from the previous version, and I have only a few minor comments and suggested edits for the authors to consider. These comments and revisions are indicated in the marked up pdf that I'm attaching to my review.

I've flagged a few places, including the article title, where adding "western boundary current" will better represent the focus and main results of this work. I have also flagged a couple of passages stating that high-resolution models will lead to better decision-making related to climate change adaptation. My comments suggest softening these statement at least a bit unless the authors can point to some concrete evidence supporting claims of increased value from increased spatial resolution in future ocean scenarios.

Overall, this manuscript will make a nice, albeit incremental, contribution to the existing literature on future MHW scenarios, and I recommend it be accepted with a request for minor revisions.

Nate Mantua
NOAA/NMFS
Santa Cruz, CA USA


Reviewer #2 (Remarks to the Author):

The authors have answered my comments in a satisfactory manner and recommend accepting the paper as is.


Reviewer #3 (Remarks to the Author):

This is the second time I review this manuscript and I am afraid that I found it more convincing the first time. The authors changed the scope of the manuscript to focus on Western Boundary Current (WBC) regions as suggested by their data. However, instead of adding value to the manuscript, the story is lost with the only key result now being that coarse (CMIP5) and high-resolution models predict different marine heatwaves characteristics in WBC systems (which has already been showed for historical periods). Due to the complex variability, the authors do not manage to provide new general insights on how different marine heatwaves are.
While the methods and data seem appropriate, the text is well written and the figures well presented, unfortunately the conclusions are a bit shallow and lack originality.
I am convinced that the authors have all the data and skills to make a significant contribution, but more analysis is necessary. The spatial variability shown in figures 5-6 is really interesting but and too descriptive. To me, the fact that conclusions are not clear means that further investigation is required before publication. Therefore I recommend major revisions.

**Author rebuttal, second version:**

REVIEWERS' COMMENTS:

Reviewer #1 (Remarks to the Author):

The revised manuscript is much improved from the previous version, and I have only a few minor comments and suggested edits for the authors to consider. These comments and revisions are indicated in the marked up pdf that I'm attaching to my review. 

I've flagged a few places, including the article title, where adding "western boundary current" will better represent the focus and main results of this work. I have also flagged a couple of passages stating that high-resolution models will lead to better decision-making related to climate change adaptation. My comments suggest softening these statement at least a bit unless the authors can point to some concrete evidence supporting claims of increased value from increased spatial resolution in future ocean scenarios. 

Overall, this manuscript will make a nice, albeit incremental, contribution to the existing literature on future MHW scenarios, and I recommend it be accepted with a request for minor revisions.

Nate Mantua 
NOAA/NMFS 
Santa Cruz, CA USA

**Thanks for your positive comments on our revised manuscript and providing further suggestions for improvement. We revised the manuscript as recommended in the marked-up PDF with the following notes:**

- **Adding the words “in western boundary current regions” to the title of the manuscript is not possible due to the word limit (exceeding the 15-word limit by 3 words).**
- **Regarding the suggestion for Figure 7, we added the error bars representing the inter-model spread of the 23 CMIP5 models (standard deviations).**

Reviewer #2 (Remarks to the Author):

The authors have answered my comments in a satisfactory manner and recommend accepting the paper as is.

**Thanks for reviewing our manuscript once again.**

Reviewer #3 (Remarks to the Author):

This is the second time I review this manuscript and I am afraid that I found it more convincing the first time. The authors changed the scope of the manuscript to focus on Western Boundary Current (WBC) regions as suggested by their data. However, instead of adding value to the manuscript, the story is lost with the only key result now being that coarse (CMIP5) and high-resolution models predict different marine heatwaves characteristics in WBC systems (which has already been showed for historical periods). Due to the complex variability, the authors do not manage to provide new general insights on how different marine heatwaves are.
While the methods and data seem appropriate, the text is well written and the figures well presented, unfortunately the conclusions are a bit shallow and lack originality. 
I am convinced that the authors have all the data and skills to make a significant contribution, but more analysis is necessary. The spatial variability shown in figures 5-6 is really interesting but and too descriptive. To me, the fact that conclusions are not clear means that further investigation is required before publication. Therefore I recommend major revisions.

**Thanks for providing your valuable feedback on our revised manuscript. We agree that the scope has been narrowed down in the revised manuscript. However, as suggested by all of the three reviewers in their first round of review, we believe that doing so provides better contribution to the existing literature on projected marine heatwaves.**

**Reviewer comments, third version:**

**Author rebuttal, third version:**
